# Sex related disparities after complex percutaneous coronary interventions

**DOI:** 10.3389/fcvm.2024.1382585

**Published:** 2024-11-07

**Authors:** Alberto Alperi, Marcel Almendárez, Isaac Pascual, Rut Alvarez, Jose Luis Betanzos, Daniel Hernández-Vaquero, Raul Ptaszynski, Juan Francisco Ortiz, Cesar Moris, Pablo Avanzas

**Affiliations:** ^1^ Department of Cardiology, Heart Area, Hospital Universitario Central de Asturias, Oviedo, Spain; ^2^ Department of Cardiology, Health Research Institute of Asturias (Instituto de Iinvestigación Sanitaria del Principado de Asturias), Oviedo, Spain; ^3^ Department of Medicine, Faculty of Medicine, University of Oviedo, Oviedo, Spain; ^4^ Department of Cardiology, Centro de Investigación en Red de Enfermedades Cardiovasculares (CIBERCV), Madrid, Spain

**Keywords:** coronary artery disease, percutaneous coronary intervention, complex PCI, myocardial infarction, sex-related differences inverse probability of treatment weight

## Abstract

**Introduction:**

Complex Percutaneous coronary intervention (PCI) for the treatment of ischemic heart disease has increased significantly. We aimed to evaluate sex-related differences in patients undergoing complex PCI.

**Methods:**

single-center prospective observational study including patients undergoing complex PCI between 2017 and 2023. Baseline and procedural features, and mid-term outcomes were compared according to the gender distribution. The combined primary endpoint included stroke, myocardial infarction, need for a new coronary revascularization, and all-cause mortality. Propensity score (PS) matching with an inverse probability of treatment weight (IPW) approach was used to adjust for differences in baseline characteristics.

**Results:**

1,283 patients were included, 983 (76.6%) male and 300 (23.4%) female. Median follow-up was 2.4 (IQR: 1–3.8) years. There was a higher rate of no-reflow phenomenon (4% vs. 1.8%, *p* = 0.03) among female patients. In the overall cohort, female patients had a greater risk for the combined primary endpoint (HR 1.28, 95% CI: 1.02–1.59). In the matched cohort, female patients exhibited a higher risk for the combined primary endpoint (HR 1.23, 95% CI: 1.06–1.42), as well as for myocardial infarction (HR 1.34, 95% CI 1.03–1.75), and all-cause mortality (HR 1.21, 95% CI 1.02–1.45), and a trend towards a higher risk for the need of a new coronary revascularization (HR 1.22, 95% CI 0.92–1.61).

**Conclusions:**

in a contemporary cohort of patients undergoing complex PCI procedures, female patients are associated with a higher risk of early complications.

## Introduction

Cardiovascular disease continues to be the leading cause of morbidity and mortality among Western countries, and an exponential growth in its incidence is expected over the following decades due to the aging population, the increased incidence of obesity and other metabolic disorders, and certain social conditions (1). Percutaneous coronary intervention (PCI) for treating ischemic heart disease has increased dramatically over the last decades due to its low invasiveness and improved clinical outcomes (2). However, certain features of the coronary lesions are known to represent a major challenge for the interventionalists and might impact early- and long-term cardiovascular outcomes. Nowadays, the widely known complex PCI and high-risk PCI procedures have gained relevance given their frequency in clinical practice, and this has been a matter of study in recent years (3, 4).

It is broadly known that females with cardiovascular disease exhibit differential clinical features than males. For instance, women with diabetes show a greater risk for cardiovascular complications than their male counterparts, probably related with inflammatory parameters and smaller coronary vessel size (5). Additionally, their clinical course differs from men in several cardiovascular pathologies (6–8).

Little is known so far about sex-related differences in patients undergoing complex PCI, but some reports suggest there might be relevant disparities. Prior studies on patients undergoing rotational atherectomy or chronic total occlusion PCI found that women more often experience coronary dissection, cardiac tamponade, and significant complications than men (9–11). Therefore, we aimed to evaluate differences in baseline clinical conditions and procedural features among patients undergoing complex PCI procedures according to their gender. Besides, we sought to evaluate a sex-related clinical impact beyond other clinical conditions in this specific setting.

## Methods

### Patient selection

This was a single-center, observational, and prospective study. All patients receiving a complex percutaneous coronary intervention in our center between 2017 and 2023 were prospectively included in a dedicated database. Baseline and procedural features were incorporated at the time of PCI, and in-hospital outcomes were added at the time of discharge. The treating physicians decided to undergo complex PCI after carefully considering all available alternatives. Patients were informed and consented to the procedure and data collection. The local Ethics Committee of the center approved data collection and reporting (2020.026).

### Inclusion criteria

Complex PCI was defined when any of the following conditions were present (12): true bifurcation lesions according to the Medina classification (13) with a side-branch diameter of at least 2.5 mm; a chronic total occlusion; unprotected left main coronary artery disease; long coronary-artery lesions requiring at least 60 mm of stent length; multivessel PCI involving the 3 major epicardial coronary arteries being treated at the same time; a severely calcified lesion needing plaque modification with either rotational atherectomy or intravascular lithotripsy; PCI in a prior saphenous bypass graft; PCI in a single remaining patent vessel; or aorto-ostial lesions of a major epicardial coronary artery.

### Exclusion criteria

For the purpose of homogeneity, those patients presenting with cardiogenic shock that needed emergent placement of a left ventricular assist device were excluded from the present analysis.

Therefore, emergent cases (ST-elevation myocardial infarction) were included if they fulfilled the criteria for complex PCI and did not require an acute ventricular assist device placement due to cardiogenic shock.

### Follow-up

Clinical follow-up was performed 6 months after the intervention and yearly thereafter. All relevant clinical events were updated at every outpatient visit. In case of missing a clinical follow-up, telephonic contact and a review of the medical files were performed to ensure live status and to avoid missing any relevant clinical event for every patient. In our region, healthcare professionals have unrestricted access to the entire clinical history of the patient, hence minimizing the miss of any significant event.

### Endpoints

The main combined primary endpoint included stroke, admission due to myocardial infarction, the need for a new coronary revascularization, and all-cause mortality.

Secondary endpoints were the individual components of the main primary endpoint.

### Statistical analysis

Results are displayed as numbers (percentage) for categorical data and as mean (standard deviation) for continuous variables. Student's *t*-test was used to compare normally continuous variables and the Mann–Whitney *U*-test was used for continuous non-normally distributed variables. The chi squared and Fisher's exact tests were used to compare categorical variables. Two groups according to patient's gender (male and female) were used to evaluate baseline characteristics and outcomes.

Propensity score (PS) matching with an inverse probability of treatment weight (IPW) approach was used to adjust for differences in baseline characteristics and potential confounders. A PS was obtained for each patient to estimate the propensity toward belonging to a specific group (female vs. male). This was done by means of a multivariate logistic regression including the following covariates: age, diabetes mellitus, hypertension, chronic kidney disease, peripheral artery disease, history of stroke, left ventricular ejection fraction (LVEF), clinical presentation (stable coronary disease, unstable angina, non-ST elevation myocardial infarction or ST elevation myocardial infarction), PCI to a chronic total occlusion lesion, PCI to a true bifurcation lesion, PCI of an aorto-ostial lesion, PCI involving the left main coronary artery, severe calcification of the treated coronary vessel, and the use of an intra-aortic balloon pump during PCI. The propensity score calculation allowed case-weight estimation to predict the inverse probability of belonging to the female group among the study participants. The case weights balanced the cohorts for an IPW analysis that included all patients with available data for the variables included in the propensity model. The adequate balancing of covariate distribution between the matched groups was numerically assessed by means of standardized means differences after IPW-matching, and graphically assessed by means of the cumulative probability plots for raw and IPW-adjusted data (Supplementary Figure S1). Then, an inverse probability of treatment-weighted Cox regression was performed to determine the relation between gender and our primary and secondary endpoints. Survival-free curves for both groups are displayed using the Kaplan–Meier method. Data analyses were performed using STATA (v14.0; StataCorp), and *p*-values <0.05 were considered significant.

## Results

A total of 1,283 patients were included, 983 (76.6%) male and 300 (23.4%) female. The main baseline characteristics of the study population are presented in Table 1. Female patients exhibited an older age at the time of PCI (73.8 ± 11.1 vs. 69.6 ± 11.4 years, *p* < 0.001), had a greater LVEF (52.8 ± 11% in females vs. 50.9 ± 11.7% in males, *p* = 0.02), and a trend towards a higher rate of left main PCI and a higher rate of aorto-ostial lesions (28.3% female vs. 21.7% male, *p* = 0.02). Male patients exhibited a higher burden of peripheral artery disease (18.4% male vs. 8.3% female, *p* = 0.001).

**Table 1:** Baseline characteristics according to gender in the overall cohort.

	Men (*n* = 983)	Women (*n* = 300)	*p* value
Age, years	69.6 ± 11.4	73.8 ± 11.1	0.001
Diabetes Mellitus	367 (33.3)	109 (36.3)	0.75
Hypertension	681 (69.3)	214 (71.3)	0.5
Dyslipidemia	621 (63.2)	185 (65.7)	0.64
Chronic kidney disease	182 (14.5)	54 (18.1)	0.85
Prior MI	42 (4.3)	10 (3.3)	0.62
Prior CABG	79 (8)	16 (5.3)	0.12
Peripheral artery disease	181 (18.4)	25 (8.3)	0.001
Prior Stroke	85 (8.6)	18 (6)	0.14
Presentation:			0.18
Stable CAD	375 (38.2)	94 (31.3)	
Unstable angina	152 (15.5)	53 (17.7)	
NSTEMI	274 (27.9)	89 (29.7)	
STEMI	182 (18.5)	64 (21.3)	
LVEF, %	50.9 ± 11.7	52.8 ± 11.5	0.02
Chronic total occlusion	159 (16.2)	42 (14)	0.36
Number of diseased vessels	1.62 ± 0.8	1.58 ± 0.8	0.43
Number of treated vessels	1.30 ± 0.6	1.31 ± 0.6	0.82
Left main PCI	379 (38.6)	132 (44)	0.09
Bypass graft PCI	24 (2.4)	8 (2.7)	0.83
3-vessel PCI	42 (4.3)	13 (4.3)	0.97
Aorto-ostial lesion PCI	213 (21.7)	85 (28.3)	0.02
Bifurcation lesion PCI	320 (30.7)	91 (30.3)	0.44
Euroscore 2	4.7 ± 3.1	4.9 ± 4.6	0.39

CABG, coronary artery bypass graft; CAD, coronary artery disease; LVEF, left ventricle ejection fraction; MI, myocardial infarction; NSTEMI, non-ST elevation myocardial infarction; PCI, percutaneous coronary intervention; STEMI, ST elevation myocardial infarction.

The main procedural features are displayed in Table 2. The main access was radial (61% in males and 55.5% in females). The rates of true bifurcation lesions, severe lesion angulation, and severe calcification were comparable between genders, but there was a trend towards a greater use of intracoronary lithotripsy (9.4% vs. 6%, *p* = 0.07), and higher number of stents and stent length among male PCI recipients.

**Table 2:** Procedural and in-hospital characteristics according to gender distribution in the overall cohort.

	Men (*n* = 983)	Women (*n* = 300)	*p* value
Access			0.17
Radial	596 (61)	166 (55.5)	
Femoral	371 (37.9)	131 (43.8)	
Double access	11 (1.1)	2 (0.7)	
Bifurcation strategy:			0.71
Provisional-stent	192 (62.9)	51 (60.7)	
Double-stent	113 (37.1)	33 (39.3)	
Severe angulation	74 (7.5)	19 (6.3)	0.49
Severe calcification	316 (32.2)	102 (34)	0.55
Use of cutting or scoring-balloon	135 (13.8)	33 (11)	0.21
Use of drug-coated balloon	217 (22.3)	43 (14.4)	0.003
Use of intracoronary lithotripsy	92 (9.4)	18 (6)	0.07
Use of rotablation	140 (14.2)	41 (13.7)	0.80
Intra-aortic balloon pump	107 (10.9)	43 (14.3)	0.11
Number of stents	2.17 ± 1.2	2.03 ± 1.2	0.08
Length of stent	53 ± 31	47.8 ± 28	0.01
Time of fluoroscopy	27.5 ± 89	22.7 ± 20	0.37
Dose of radiation	3,190 ± 3600	2,330 ± 1842	0.001
Contrast media used	191 ± 96	178 ± 88	0.06
Procedural complications			
Unsuccessful PCI	49 (4.9)	12 (4)	0.48
Vascular closure failure	10 (1)	2 (0.7)	0.58
Perforation	9 (0.9)	6 (2)	0.13
Dissection	39 (4)	13 (4.3)	0.78
Side branch closure	13 (1.3)	6 (2)	0.40
No-reflow	18 (1.8)	12 (4)	0.03
Procedural death	11 (1.1)	8 (2.7)	0.05
In hospital complications			
Vascular complication	30 (3)	13 (4.3)	0.28
Stroke/TIA	12 (1.2)	3 (1)	0.76
Contrast-induced nephropathy	98 (10)	33 (11)	0.61
In-hospital death	79 (8)	35 (11.7)	0.05
Length of hospitalization	7 ± 9.7	7.9 ± 12	0.19

PCI, percutaneous coronary intervention; TIA, transient ischemic attack.

Procedural-related complications and in-hospital outcomes are summarized in Table 2. There were no significant differences between study groups in PCI failure (4.9% vs. 4%). However, there was a higher rate of no-reflow phenomenon (4% vs. 1.8%, *p* = 0.03) and a trend towards a higher risk of procedural mortality (2.7% vs. 1.1%, *p* = 0.05) among female patients. Similarly, differences in in-hospital complications were not statistically significant but for a trend towards a higher risk of in-hospital mortality for the female group.

Median follow-up was 2.4 (IQR: 1–3.8) years. Over that period, a total of 290 (22.6%) patients died (205 [20.8%] and 85 [28.3%] deaths among male and female patients, respectively), 14 (1.1%) patients had a stroke (8 [0.8%] and 6 [2%] strokes among male and female patients, respectively), 106 (8.3%) patients underwent new coronary revascularizations (81 [8.2%] and 25 [8.3%] new coronary revascularizations among male and female patients, respectively), and there were 116 (9.1%) admissions for acute myocardial infarction (87 [8.8%] and 29 [9.7%] among male and female patients, respectively).

**Figure 1:**
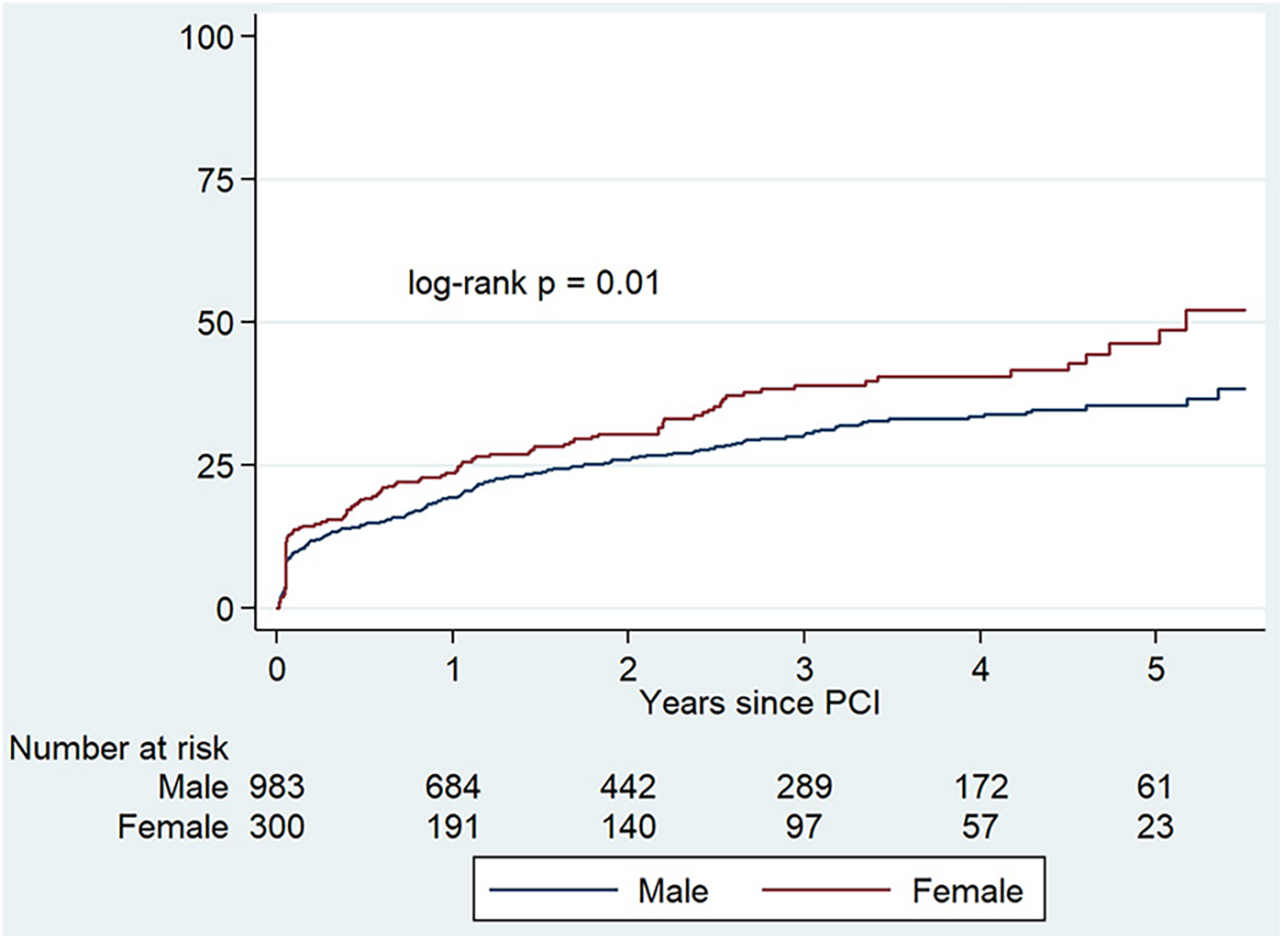
Kaplan–Meier cumulative incidence estimates for the combined primary endpoint (stroke, new coronary revascularization, myocardial infarction or all-cause mortality) in the overall cohort of patients.

**Figure 2:**
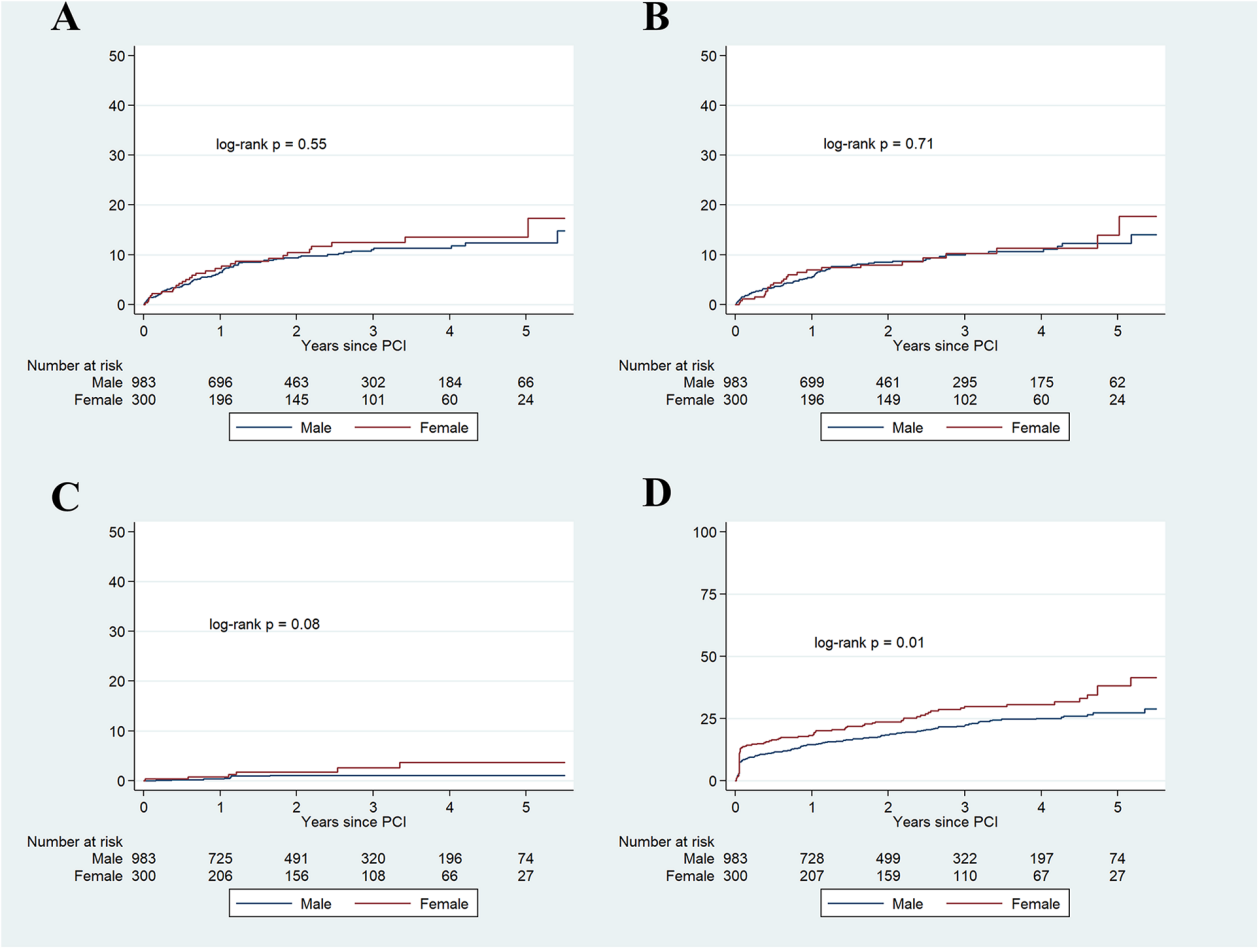
Kaplan–Meier cumulative incidence estimates for myocardial infarction **(A)**, new coronary revascularization **(B)**, stroke **(C)**, and all-cause mortality **(D).**

In the univariate Cox regression analysis, several variables conferred a significantly higher risk for the primary combined endpoint (older age, diabetes, hypertension, chronic kidney disease, peripheral artery disease, number of diseased vessels, left main PCI, and true bifurcation lesions), whereas other were protective factors (higher LVEF and PCI of a total chronic occlusion). Besides, female gender was also associated with a higher risk for the combined endpoint (HR 1.32, 95% CI: 1.07–1.64). In the multivariate analysis, after adjustment for covariates, female gender was yet associated with a higher risk of death, stroke, myocardial infarction or new coronary revascularization (HR 1.28, 95% CI: 1.02–1.59) (Table 3). This higher risk for the combined endpoint was mainly based on all-cause mortality (HR 1.38, 95% CI: 1.07–1.77) and stroke rates (HR 2.57, 95% CI: 0.89–7.4), and no significant differences for myocardial infarction or new revascularization were observed for the overall unadjusted cohort (Table 4). Kaplan–Meier graphics for the primary and secondary endpoints are represented in Figures 1, 2, respectively.

**Table 3:** Univariate and multivariate cox regression analysis for the primary combined endpoint of myocardial infarction, stroke, new coronary revascularization and all-cause mortality.

Variable	Univariate		Multivariate	
	HR (95% CI)	*p* value	HR (95% CI)	*p* value
Age, years	1.03 (1.02–1.04)	0.001	1.02 (1.01–1.03)	0.001
Diabetes Mellitus	1.82 (1.48–2.22)	0.001	1.67 (1.32–2.09)	0.001
Hypertension	1.32 (1.05–1.66)	0.02	1.02 (0.78–1.36)	0.83
Dyslipidemia	1.11 (0.90–1.37)	0.32		
Chronic kidney disease	2.2 (1.76–2.7)	0.001	1.26 (0.96–1.64)	0.09
Prior MI	1.39 (0.90–2.14)	0.13		
Prior CABG	1.19 (0.82–1.72)	0.35		
Peripheral artery disease	1.84 (1.44–2.36)	0.001	1.46 (1.09–1.94)	0.009
Prior Stroke	1.54 (1.1–2.14)	0.01	1.02 (0.71–1.49)	0.89
Presentation:				
Stable CAD (reference)				
Unstable angina	1.27 (0.92–1.77)	0.14	1.03 (0.73–1.48)	0.83
NSTEMI	1.85 (1.42–2.4)	0.001	1.27 (0.95–1.68)	0.10
STEMI	2.12 (1.61–2.79)	0.001	1.57 (1.13–2.17)	0.006
LVEF, %	0.96 (0.95–0.97)	0.001	0.97 (0.96–0.98)	0.001
Chronic total occlusion	0.35 (0.26–0.52)	0.001	0.64 (0.42–0.98)	0.04
Number of diseased vessels	1.43 (1.27–1.61)	0.001	1.27 (1.11–1.44)	0.001
Left main PCI	1.74 (1.42–2.12)	0.001	1.09 (0.83–1.44)	0.53
Bypass graft PCI	1.99 (1.21–3.29)	0.007	2.01 (1.16–3.45)	0.02
3-vessel PCI	1.37 (0.89–2.11)	0.15		
Aorto-ostial lesion PCI	1.21 (0.97–1.53)	0.09	1.12 (0.87–1.44)	0.39
Bifurcation lesion PCI	1.59 (1.29–1.96)	0.001	1.33 (1.01–1.74)	0.03
Gender (female vs. male)	1.32 (1.07–1.64)	0.01	1.28 (1.02–1.59)	0.04

CABG, coronary artery bypass graft; CAD, coronary artery disease; CI, confidence interval; HR, hazard ratio; LVEF, left ventricle ejection fraction; MI, myocardial infarction; NSTEMI, non-ST elevation myocardial infarction; PCI, percutaneous coronary intervention; STEMI, ST elevation myocardial infarction.

**Table 4:** Survival analysis for the primary and secondary endpoints according to gender in the overall and IPW-adjusted populations.

Overall cohort		
Endpoint	HR (95% CI)	*p* value
Combined primary endpoint	1.32 (1.07–1.64)	0.01
Stroke	2.57 (0.89–7.4)	0.08
New revascularization	1.06 (0.68–1.67)	0.79
Myocardial infarction	1.13 (0.75–1.73)	0.55
All-cause mortality	1.38 (1.07–1.77)	0.01
IPW-adjusted cohort		
Endpoint	HR (95% CI)	*p* value
Combined primary endpoint	1.23 (1.06–1.42)	0.01
Stroke	1.71 (0.77–3.84)	0.19
New revascularization	1.22 (0.92–1.61)	0.17
Myocardial infarction	1.34 (1.03–1.75)	0.03
All-cause mortality	1.21 (1.02–1.45)	0.03

CI, confidence interval; HR, hazard ratio; IPW, inverse probability of treatment weight.

### IPW-adjusted cohort

After PS-IPW adjustment, baseline characteristics were well-balanced between study groups (Supplementary Tables S1 and S2 and Supplementary Figure S1).

**Figure 3:**
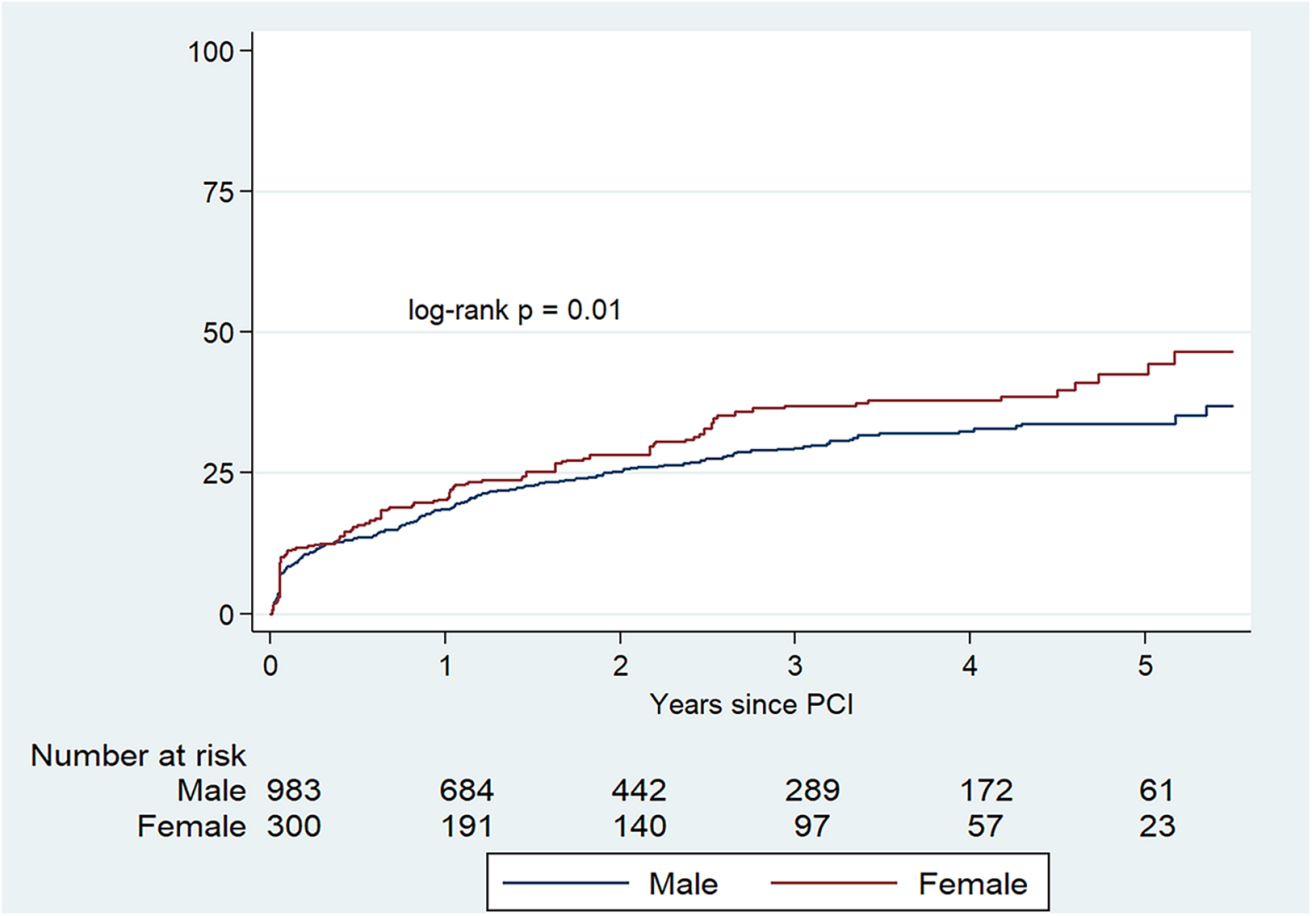
Kaplan–Meier cumulative incidence estimates for the combined primary endpoint (stroke, new coronary revascularization, myocardial infarction or all-cause mortality) in the IPW-adjusted cohort.

In the IPW-adjusted cohort, there was a higher risk for the combined primary endpoint (HR 1.23, 95% CI: 1.06–1.42) among female patients (Table 4 and Figure 3).

**Figure 4:**
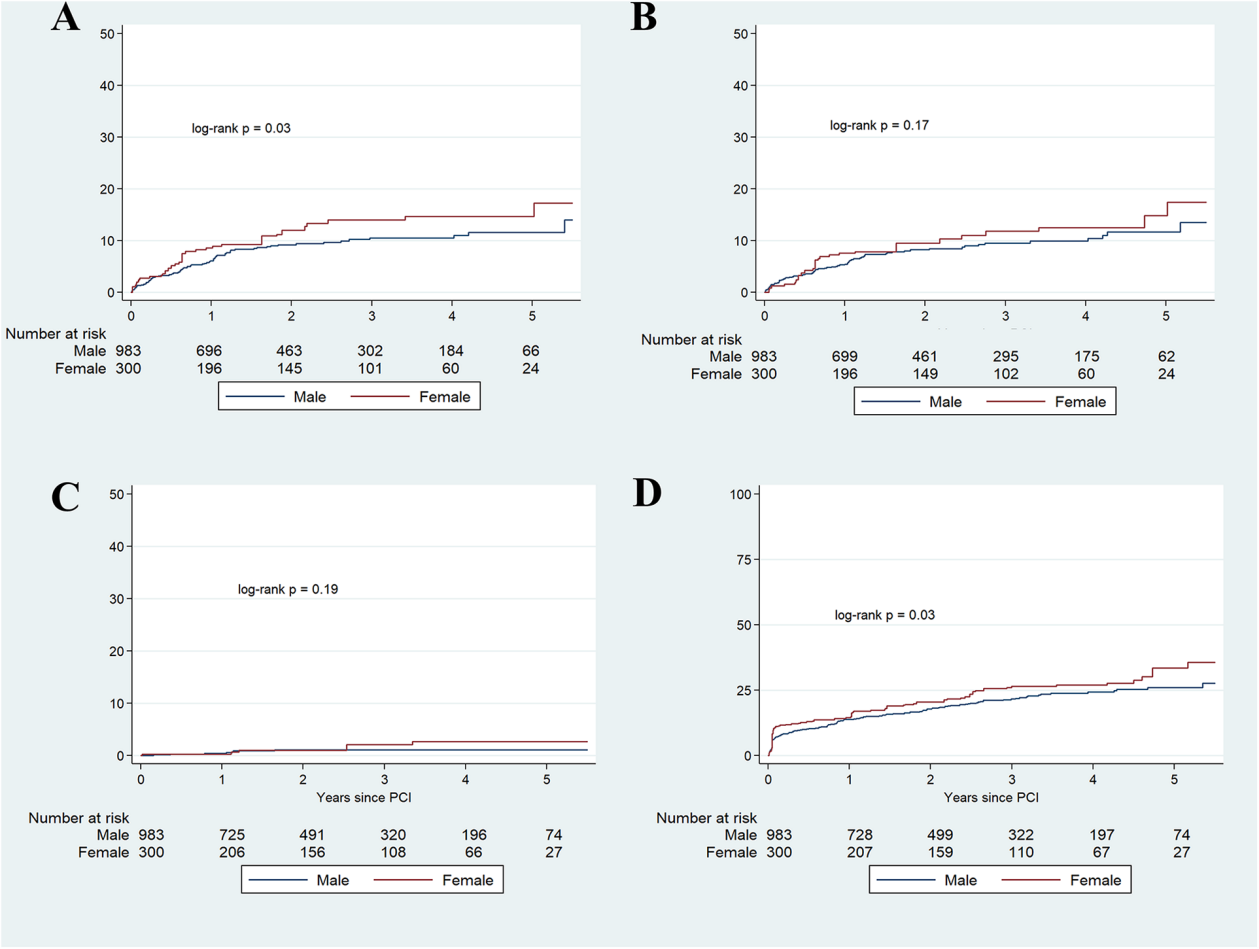
Kaplan–Meier cumulative incidence estimates for myocardial infarction **(A)**, new coronary revascularization **(B)**, stroke **(C)**, and all-cause mortality **(D)** in the IPW-adjusted cohort.

Besides, female patients exhibited higher rates for admission due to myocardial infarction (HR 1.34, 95% CI: 1.03–1.75), a higher risk for all-cause mortality (HR 1.21, 95% CI: 1.02–1.45), and a trend towards a higher risk for the need of a new coronary revascularization (HR 1.22, 95% CI: 0.92–1.61) (Table 4 and Figure 4).

## Discussion

The main findings of our investigation are summarized as follows: (i) female patients undergoing complex PCI exhibited differential features compared to their male counterparts, like a higher rate of left main PCI, aorto-ostial lesion PCI, and a higher risk for non-reflow phenomenon. (ii) female gender was independently associated with a higher risk for the combined primary endpoint (stroke, myocardial infarction, new coronary revascularization, and all-cause mortality) over follow-up, and those results remain concordant after adjustment for baseline covariates; (iii) in the adjusted cohort, female patients exhibited a higher risk for myocardial infarction and a trend towards a higher risk for new coronary revascularization over follow-up.

### Sex-related differences in baseline and procedural characteristics

Although cardiovascular disease develops 5 to 10 years later in women than in men, this is yet the leading cause of death in women older than 65 years. In line with prior data, the higher age of women presenting with ischemic heart disease was also manifest in our patient cohort. Despite this age difference, male patients carried a slightly higher cardiovascular disease burden in our patient population, with a significantly higher rate of peripheral artery disease and comparable rates of diabetes, hypertension, and chronic kidney disease.

Coronary artery disease could be underrecognized among female patients, given the disparities in clinical presentation (frequent association of atypical symptoms) and the perception that women are invariably more protected against cardiovascular disease due to inherent endocrine characteristics (e.g., estrogen-dependent vasodilatation of the endothelial wall). These facts could lead to a delayed diagnosis among women, causing a more advanced coronary disease at presentation and ultimately impacting prognosis.

Notably, there was a lower use of contemporary techniques for the management of complex coronary lesions in women than in men in our cohort. As a matter of fact, despite similar rates of severe lesion calcification, the use of intravascular lithotripsy and cutting/scoring-balloon was numerically lower among female patients. These data are in line with prior reports in which ischemic stress testing and invasive procedures for the diagnosis and management of ischemic heart disease were underused in women (14). However, it should be noted that coronary anatomy and pathophysiology vary significantly between genders. Women tend to present with a smaller vessel size, less collateral flow, lower coronary flow reserve, greater vascular stiffness, and more tortuous anatomies (15). Hence, whether the disparities observed in PCI technical aspects are driven by pathophysiological and anatomical features between men and women remains widely unknown. Nevertheless, in our cohort, there was a higher risk for no-reflow in women even after adjustment by baseline characteristics, which may underline the more complex vessel and endothelial regulation associated with the female gender (7). This fact might also play a role in the higher rate of complications observed in women undergoing rotational and orbital atherectomy (9, 10, 16, 17). It could be a factor underlying the numerically higher rate of early mortality (both procedural and in-hospital) observed in our investigation.

In the setting of left-main PCI, women have frequently been underrepresented in clinical trials. For instance, in the SYNTAX trial, women with unprotected left main disease accounted for only 10.3% of participants. Besides, women within the PCI group had a higher adjusted 4-year mortality rate than men (18). Noteworthy, there were both a higher rate of left main PCI and of aorto-ostial lesions among women in our patient population. Considering that women develop ischemic heart disease at an older age than men and that cardiac surgical risk increases steadily with age, those facts might translate into a greater proportion of women with left main disease undergoing PCI rather than surgery. Hence, aorto-ostial lesions involving the left main ostium were probably more frequently managed percutaneously among the female subgroup. Besides, bifurcation involvement within the left main was probably less frequent in women, rarely needing two-stent strategies in such a relevant anatomic position. It should be also mentioned that ostial lesions (more frequent among women) are characterized by being more calcific and rigid, often associating a recoil phenomenon after stent placement, altogether increasing PCI complexity (19).

Noteworthy, women presented more frequently exhibiting an acute myocardial infarction. This issue might be related to differences in clinical presentation, as women more frequently experienced atypical symptoms such as weakness, palpitations, or lightheadedness, hence delaying the diagnosis of ischemic heart disease until most severe settings (STEMI) developed.

### Sex-related differences for the study endpoints

Although riskier baseline conditions may partially account for poorer outcomes among the female group of patients, the concordant results in both the multivariate analysis and the IPW-adjusted cohort emphasize the differential clinical evolution for both genders beyond baseline features. In the context of an all-comer PCI study, it has already been reported that women carried a higher early and mid-term risk for major cardiovascular outcomes than men (20). The main reasons postulated behind this finding were the low inclusion of women in randomized trials, resulting in device-based techniques being optimized for men, as well as the greater rates of in-stent restenosis and lesion progression linked to the small diameter of target vessels. These facts could be even more accentuated in our patient population, given the greater complexity of the coronary anatomy compared to prior reports on all-comer PCI procedures. Consequently, the relevance of any technical aspect potentially impacting clinical outcomes (e.g., rigorous lesion preparation and optimal treatment of smaller vessels) increases in parallel with coronary lesion complexity.

As mentioned above, the well-known delay between symptom onset and diagnosis of ischemic heart disease in the female gender could also be a potential source for unequal clinical findings (21). Since the time between ischemic symptoms onset and reperfusion are key prognostic factors both in ST elevation and non-ST elevation myocardial infarction, a delayed presentation in women in our cohort could partially explain their poorer prognosis. Unfortunately, we do not have systematic data for all patients on the time interval between anginal symptoms onset and the time of PCI, so this matter still needs further investigation.

In light of our results, further investigations purposely designed to assess the net benefit of complex PCI procedures among women are needed, along with studies that implement strategies to mitigate the residual risk that female patients face after a PCI procedure beyond any disparities in baseline conditions.

### Limitations

This study presents several limitations. Firstly, its observational nature is more prone to bias than randomized studies. Secondly, despite the good comparability between groups after IPW adjustment, the presence of unnoticed unbalanced confounders could not be discarded. Thirdly, some variables that might be of relevance were not assessed in our database, such as the SYNTAX score. Social biases in the access to medical care cannot be fully excluded, but its impact might be marginal considering that the study was developed in a region where universal health-care coverage without risking financial hardship has been implemented for a long period of time. Finally, recent trials have demonstrated better clinical outcomes in complex PCI settings such as bifurcation lesions with the guidance of intravascular imaging (22, 23), but we did not use systematic intracoronary imaging in our series. Future studies should further explore this issue.

In conclusion, in a contemporary cohort of patients undergoing complex PCI procedures, female patients are associated with a higher risk of early complications and receive less frequently state-of-the-art intravascular techniques despite similar complexity. Additionally, after adjustment for baseline features, women exhibited a higher risk for all-cause mortality, myocardial infarction, and for the combined primary endpoint of stroke, coronary revascularization, myocardial infarction, and all-cause mortality at mid-term follow-up.

## Data Availability

The raw data supporting the conclusions of this article will be made available by the authors, without undue reservation.
