# Supplementary material for: Sex related disparities after complex percutaneous coronary interventions
Source: Front Cardiovasc Med. 2024 Nov 7;11:1382585. doi: 10.3389/fcvm.2024.1382585 (PMC11578988; doi:10.3389/fcvm.2024.1382585)

Supplementary Table 1: Baseline characteristics after IPW-matching according to gender distribution.

	Male (n=973)	Female (n=298)	p value	Std. mean difference
Age, years	70.65±11.3	70.3±11.7	0.64	-0.03
Diabetes Mellitus	363 (37.3)	107 (35.9)	0.66	-0.02
Hypertension	676 (69.5)	208 (70)	0.75	0.01
Dyslipidemia	613 (63)	190 (63.8)	0.80	0.01
Chronic kidney disease	179 (18.4)	51 (17.1)	0.61	-0.03
Prior MI	41 (4.2)	11 (3.7)	0.65	-0.04
Prior CABG	76 (7.8)	21 (7.1)	0.69	-0.03
Peripheral artery disease	157 (16.1)	50 (16.7)	0.81	0.01
Prior Stroke	81 (8.3)	21 (7)	0.47	-0.05
Presentation:			0.33	
Stable CAD	358 (36.8)	100 (33.6)		
Unstable angina	150 (15.4)	54 (18.1)		
NSTEMI	278 (28.6)	94 (31.5)		
STEMI	187 (19.2)	50 (16.8)		
LVEF	51.5±11.2	51.6±11.8	0.92	0.01
Chronic total occlusion	151 (15.5)	47 (15.8)	0.91	0.01
Number of diseased vessels	1.63±0.8	1.59±0.8	0.48	-0.08
Number of treated vessels	1.31±0.6	1.30±0.6	0.75	-0.03
Left main PCI	386 (39.7)	113 (37.9)	0.58	-0.04
Bypass graft PCI	24 (2.48)	8 (2.73)	0.77	0.03
3-vessel PCI	42 (4.3)	12 (4)	0.82	-0.01
Aorto-ostial lesion PCI	215 (22.1)	72 (24.1)	0.47	0.05
Bifurcation lesion PCI	312 (32.1)	91 (30.5)	0.61	-0.03

CABG: coronary artery bypass graft. CAD: coronary artery disease. LVEF: left ventricle ejection fraction. MI: myocardial infarction. NSTEMI: non-ST elevation myocardial infarction. PCI: percutaneous coronary intervention. STEMI: ST elevation myocardial infarction. STD: standardized.

Supplementary Table 2: Procedural and in-hospital characteristics according to gender distribution in the IPW-adjusted cohort.

	Men (n=973)	Women (n=298)	p value
Access			0.16
Radial	587 (60.5)	163 (54.7)	
Femoral	372 (38.5)	132 (44.5)	
Double access	10 (1)	2 (0.8)	
Bifurcation strategy:			0.86
Provisional-stent	197/318 (62)	55/88 (62.6)	
Severe angulation	71 (7.3)	20 (6.7)	0.72
Severe calcification	315 (32.4)	102 (34.2)	0.56
Use of cutting or scoring-balloon	134 (13.8)	31 (10.5)	0.14
Use of intracoronary lithotripsy	90 (9.3)	20 (6.7)	0.16
Use of rotablation	140 (14.4)	45 (15.1)	0.76
Intra-aortic balloon pump	113 (11.6)	34 (11.4)	0.93
Number of stents	2.18±1.2	2.01±1.17	0.06
Length of stent	52.7±30.9	48.7±27.6	0.05
Time of fluoroscopy	27.9±98	23.2±18	0.40
Dose of radiation	3150±3200	2320±1715	0.01
Contrast media used	190±94	186±91	0.52
Procedural complications			
Unsuccessful PCI	48 (4.9)	14 (4.8)	0.92
Vascular closure failure	9 (0.9)	2 (0.7)	0.81
Perforation	10 (1.1)	6 (2)	0.23
Dissection	38 (3.9)	11 (3.7)	0.87
Side branch closure	13 (1.3)	5 (1.7)	0.61
No-reflow	18 (1.9)	11 (3.7)	0.07
Procedural death	12 (1.2)	7 (2.3)	0.17
**In-hospital outcomes**			
Vascular complication	31 (3.2)	14 (4.7)	0.23
Stroke/TIA	11 (1.2)	3 (1)	0.77
Contrast-induced nephropathy	98 (10.1)	33 (11.1)	0.62
In-hospital death	80 (8.2)	30 (10.1)	0.31
Length of hospitalization	7.1±9.6	7.9±11.9	0.23

PCI: percutaneous coronary intervention. TIA: transient ischemic attack.

Supplementary Figure 1: cumulative probability of the propensity score values for the overall population (unmatched) and the PS-IPW-adjusted cohort.

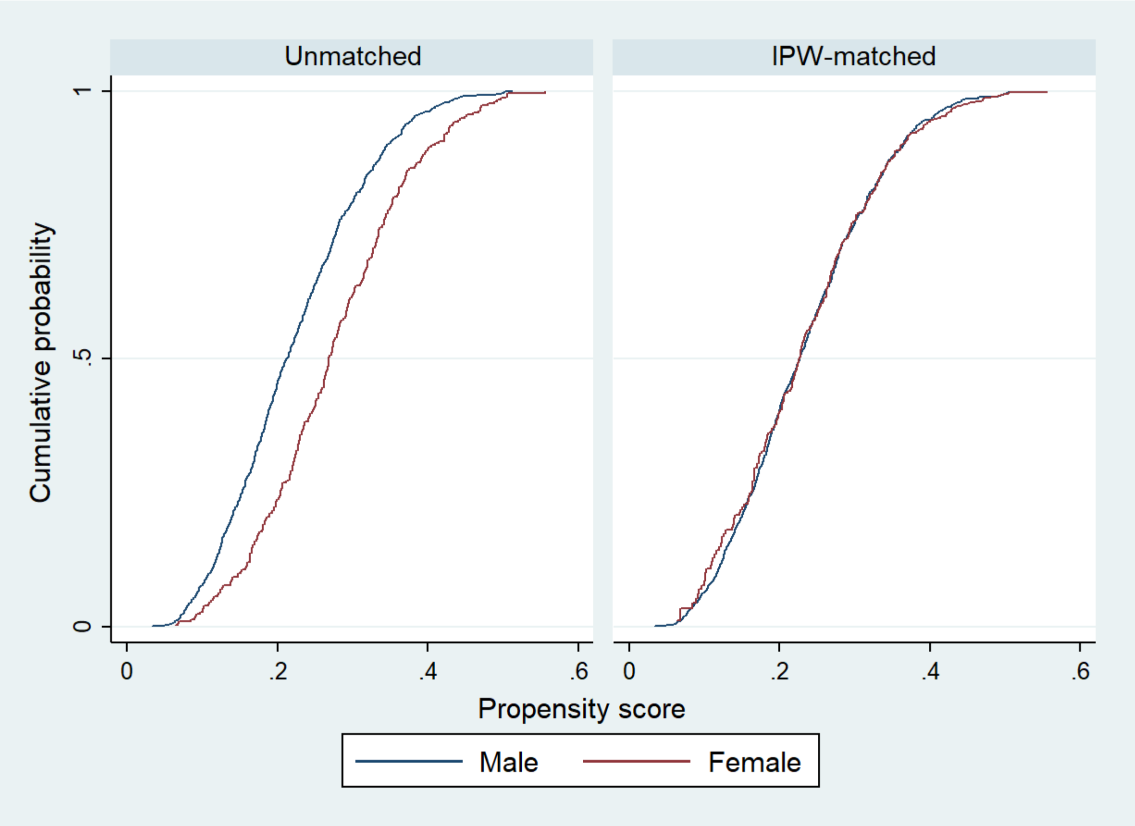

Supplement: Supplementary file 1 [file Datasheet1.pdf]
